# Baseline systemic immune-inflammation index and a novel FLIPI-SII model predict POD24 and long-term survival in grade 1–3a follicular lymphoma

**DOI:** 10.3389/fimmu.2026.1869187

**Published:** 2026-07-23

**Authors:** Jingwei Yu, Cong Sun, Qian Liu, Ganggang Wang, Jiesong Wang, Lanfang Li, Lihua Qiu, Zhengzi Qian, Shiyong Zhou, Xianhuo Wang, Huilai Zhang

**Affiliations:** 1 State Key Laboratory of Druggability Evaluation and Systematic Translational Medicine/Department of Lymphoma, Tianjin Medical University Cancer Institute and Hospital, National Clinical Research Center for Cancer, Tianjin’s Clinical Research Center for Cancer, Key Laboratory of Cancer Prevention and Therapy, The Sino-US Center for Lymphoma and Leukemia Research, Tianjin, China; 2 First Teaching Hospital of Tianjin University of Traditional Chinese Medicine, National Clinical Research Center for Chinese Medicine, Tianjin, China; 3 Cancer Center, Shanxi Bethune Hospital, Shanxi Academy of Medical Sciences, Third Hospital of Shanxi Medical University, Tongji Shanxi Hospital, Taiyuan, China; 4 Department of Lymphoma & Head and Neck Oncology, Clinical Oncology School of Fujian Medical University, Fujian Cancer Hospital, Fuzhou, China

**Keywords:** FLIPI-SII, follicular lymphoma, immunochemotherapy, POD24, prognostic model, risk stratification, SII, systemic immune-inflammation index

## Abstract

Follicular lymphoma (FL) exhibits substantial clinical heterogeneity, and progression of disease within 24 months (POD24) represents a critical adverse clinical endpoint. Traditional prognostic models rely predominantly on tumor-related parameters and overlook systemic immune-inflammatory status. The newly established FLIPI24 index emphasizes peripheral blood markers but lacks immune-related indicators. Here, we investigated the prognostic value of the baseline systemic immune-inflammation index (SII) and developed a novel FLIPI-SII model to improve risk stratification in patients with grade 1–3a FL. A total of 628 patients were used for model training/internal validation, and 187 R-CHOP-treated patients for external validation. High SII (>580) was significantly correlated with inferior overall survival (OS) and progression-free survival (PFS), and was identified as an independent prognostic risk factor in multivariate regression analysis. The predictive efficacy of SII remained stable in patients receiving rituximab-based immunochemotherapy. We further constructed a 6-item FLIPI-SII scoring system, which categorized patients into four distinct risk subgroups and exerted powerful predictive performance for POD24. The model effectively predicted both PFS and OS in our cohort and was validated in two external centers. The FLIPI-SII model presented higher C-index values than conventional FLIPI, FLIPI2, and PRIMPI for both OS and PFS prediction. In conclusion, baseline SII acts as a stable, non-invasive, and independent prognostic biomarker for grade 1–3a FL. The newly developed FLIPI-SII model greatly improves the prediction of POD24 and long-term survival, providing a reliable immune-associated tool for individualized clinical risk assessment.

## 1 Introduction

Follicular lymphoma (FL) accounts for approximately 20% of all non-Hodgkin lymphomas worldwide and manifests as an indolent yet incurable hematological malignancy with substantial clinical heterogeneity (1, 2). The widespread application of rituximab-based immunochemotherapy has dramatically improved long-term survival outcomes for FL patients over the past two decades (3). Nevertheless, nearly 20% of patients develop early disease progression within 24 months after frontline treatment, defined as POD24, which is closely associated with histological transformation and early mortality (4–6). Therefore, it is urgently needed to identify convenient and effective pretreatment biomarkers to screen high-risk populations and optimize individualized therapeutic strategies.

Current mainstream prognostic scoring systems, including FLIPI, FLIPI2 and PRIMPI, predominantly incorporate clinical and laboratory indicators that reflect baseline tumor burden, such as age, Ann Arbor stage and serum lactate dehydrogenase levels (7–9). However, these classic models fail to incorporate host immune and inflammatory status, which is now recognized as a core regulator of tumor microenvironment remodeling, immune escape and treatment resistance in B-cell indolent lymphomas (10). Unlike aggressive malignancies driven by rapid tumor proliferation and frequent genetic aberrations, grade 1–3a FL progresses slowly and is predominantly governed by a localized and systemic chronic, low-grade immune-inflammatory dysregulation. This reciprocal interaction between malignant B cells and the immune macroenvironment constitutes the unique pathological basis for its heterogeneous clinical course and treatment sensitivity (11).

The systemic immune-inflammation index (SII), a composite peripheral blood biomarker calculated by neutrophils, platelets and lymphocytes, can objectively reflect the balance between pro-tumor inflammation and anti-tumor immune surveillance (12). Crucially, rather than serving as a non-specific inflammatory proxy, the components of the SII mirror the specialized pathophysiological fabric of FL within its highly dependent germinal center-like niche. Absolute neutrophil expansion frequently correlates with an immunosuppressive microenvironment, where myeloid-derived cells can secrete pro-survival cytokines that sustain malignant B-cell niches. Simultaneously, elevated platelets may act as systemic shields, releasing growth factors and anti-inflammatory mediators that potentially blunt host active immune surveillance. Conversely, a relative contraction of the peripheral lymphocyte pool reflects a systemic exhaustion or spatial sequestration of functional cytotoxic T cells and immune effectors (13–15). Therefore, the homeostatic ratio defined by the SII captures the critical tipping point where pro-tumor inflammatory disruption eclipses host anti-lymphoma immunity. While the broad prognostic relevance of the SII has been described across various oncology settings (16–19), its specific biological capacity to complement traditional, tumor-centric FL staging systems remains unmapped. Specifically, rather than acting as an isolated predictor for early progression, whether this peripheral index can interact synergistically with clinical metrics to refine POD24 risk stratification remains unvalidated. Furthermore, whether this synchronized hematological ratio can potentially reflect resistance in patients undergoing rituximab-based immunotherapy warrants definitive evaluation in a large-sample, multicenter cohort.

Of note, the newly proposed FLIPI24 prognostic model focuses on peripheral blood routine parameters, highlighting the critical role of minimally invasive blood biomarkers in modern FL risk stratification (20). Differing from the hematological parameters adopted by FLIPI24, the SII does not merely aggregate isolated cell counts; instead, it integrates three distinct immunological axes into a synchronized ratio. This mathematically is proposed to capture the balance between inflammatory tumor-promotion and immune surveillance, thereby offering a superior biological reflection of the host-tumor equilibrium. Consequently, an integrated FLIPI-SII model holds the potential to provide a more comprehensive, multi-dimensional assessment of FL heterogeneity than FLIPI24, effectively coupling baseline tumor burden with the host’s systemic immune fitness.

In this large−sample retrospective cohort study with both internal and external validation, we aimed to: (1) determine the optimal baseline SII cutoff value in grade 1–3a FL; (2) validate the independent prognostic significance of SII for OS and PFS; (3) explore the predictive efficacy of SII in the rituximab-based treatment subgroup; and (4) construct and validate a novel FLIPI-SII integrated model to optimize risk stratification for POD24 and long-term survival.

## 2 Materials and methods

### 2.1 Study design and patients

**Figure 1:**
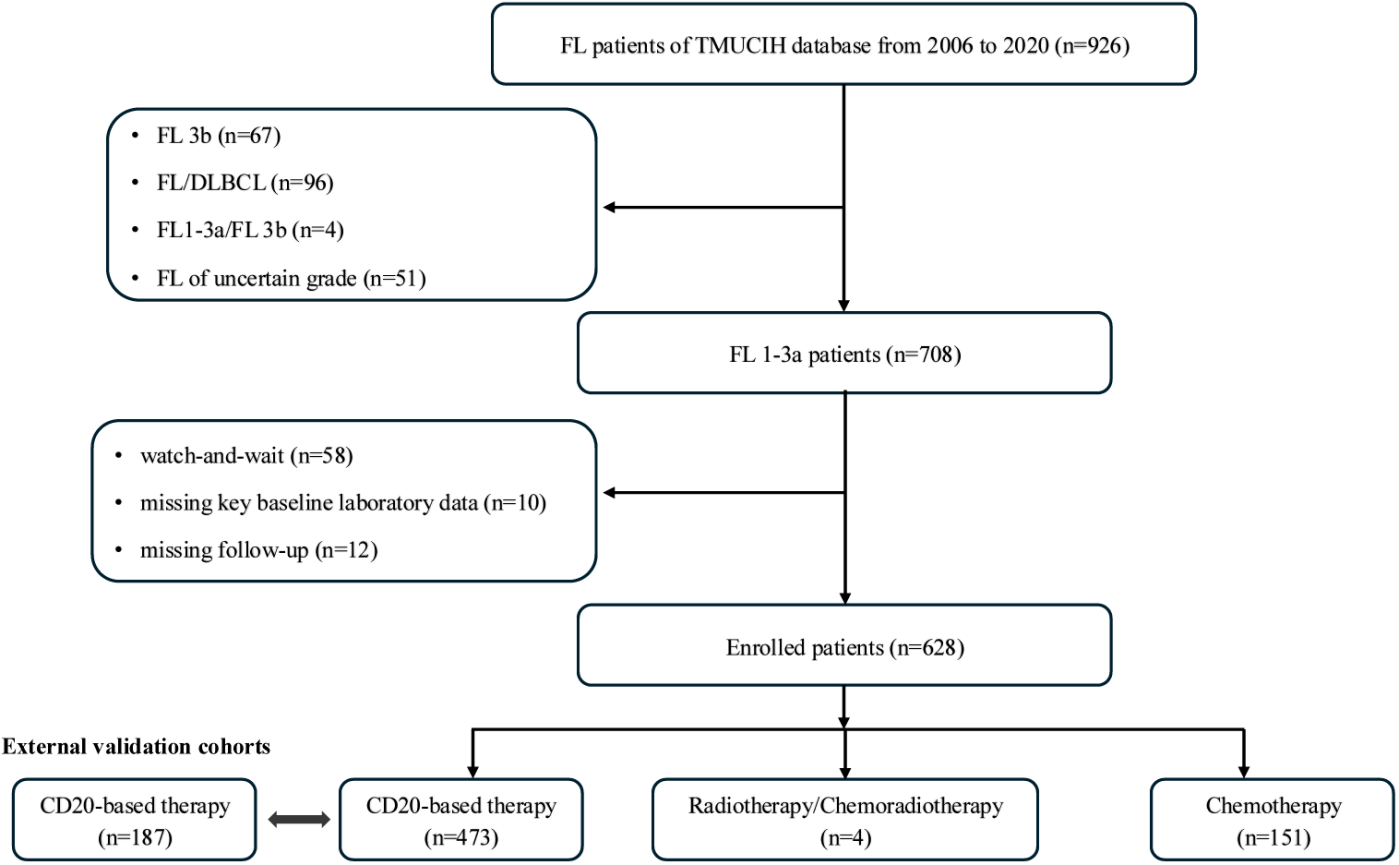
CONSORT flow diagram of patient selection. Flowchart illustrating the systematic screening and enrollment process. Out of 926 initially identified patients with follicular lymphoma (FL), 628 met the inclusion criteria (CD20+ grade 1–3a FL) and were randomly assigned to the training cohort (n = 431) and validation cohort (n = 197). An independent external validation cohort comprised 187 patients treated with R-CHOP at two additional medical centers. An independent external validation cohort comprised 187 patients treated with R-CHOP at two additional medical centers. The specific exclusion criteria and corresponding patient numbers are shown in the diagram.

This retrospective observational study consecutively enrolled newly diagnosed FL patients at Tianjin Medical University Cancer Institute and Hospital between January 2006 and December 2020. A total of 926 screened patients were initially recruited, and 628 eligible patients with grade 1–3a FL were finally enrolled after strict inclusion and exclusion criteria screening (**Figure 1**).

Inclusion criteria: (1) age ≥ 18 years; (2) histologically confirmed grade 1–3a FL according to the 2017 WHO classification with centralized pathological review; (3) complete baseline blood routine data collected within 7 days before initial treatment; (4) clear treatment indications and complete clinical follow-up data.

Exclusion criteria: (1) grade 3b FL or transformed aggressive lymphoma; (2) combined malignant tumors or severe autoimmune diseases; (3) active acute infection at diagnosis; (4) patients receiving watch-and-wait management without systematic anti-tumor treatment; (5) incomplete key clinical data or follow-up duration less than 6 months.

The Research Ethics Committee of TMUCIH and the ethics committees of the other centers reviewed and approved the study. Written informed consent was waived due to the retrospective design and the use of de-identified clinical data, in full compliance with the Declaration of Helsinki.

### 2.2 Study cohorts

All enrolled patients were randomly assigned to the training cohort (n = 431) for biomarker cutoff value screening and model construction, and an independent validation cohort (n = 197) for internal verification. Simple randomization was performed using R 4.4.1 to ensure balanced baseline characteristics between the two cohorts.

To further evaluate the generalizability of the FLIPI-SII model, we included an independent external validation cohort consisting of 187 patients with newly diagnosed follicular lymphoma treated with R-CHOP from two additional Chinese medical centers (**Figure 1**).

### 2.3 Calculation of SII and cutoff determination

Baseline peripheral blood cell counts before initial treatment were collected to calculate SII:


$$SII\;=\;Platelet\;count\;\left(\times 10^{9}/L\right)\;\times \;Neutrophil\;count\;\left(\times 10^{9}/L\right)/Lymphocyte\;count\;\left(\times 10^{9}/L\right)$$

The optimal cutoff value of SII was determined using the surv_cutpoint function from the R package survminer, which is based on maximally selected rank statistics. This method systematically evaluates all possible cutoff values within the observed range and identifies the cutoff that provides the strongest separation of survival outcomes according to the standardized log-rank statistic. To reduce the risk of selecting an unstable cutoff driven by very small subgroups, we restricted the minimum proportion of observations in each group. The raw optimal cutoff value for SII was 581.2, which was then rounded to 580 for clinical interpretability and practical application.

### 2.4 Construction of the FLIPI-SII prognostic model

The novel FLIPI-SII scoring system integrated five conventional FLIPI indicators and SII status, including age ≥60 years, Ann Arbor stage III–IV, hemoglobin<120 g/L, ≥5 involved nodal regions, elevated serum LDH, and high SII. Each positive indicator was assigned 1 point. According to the total score, patients were divided into four risk subgroups: L0 (0 points), L1–2 (1–2 points), L3–4 (3–4 points), and L5–6 (5–6 points).

### 2.5 Incremental predictive value assessment

To further evaluate the incremental predictive value of the FLIPI-SII model over established prognostic indices, the net reclassification improvement (NRI) and integrated discrimination improvement (IDI) were calculated. Time-dependent NRI and IDI analyses were performed at 3 and 5 years for OS and PFS. Considering the different predictive strengths of the existing clinical indices, FLIPI-SII was compared with FLIPI for OS prediction, whereas FLIPI-SII was compared with PRIMPI for PFS prediction. The corresponding NRI, IDI, 95% confidence intervals, and P values were calculated to quantify the improvement in risk reclassification and discrimination after incorporating SII into the prognostic model. A two-sided P value<0.05 was considered statistically significant.

### 2.6 Clinical endpoints and follow-up

The primary endpoints were OS and PFS. OS was defined as the interval from diagnosis to all-cause death or the last follow-up. PFS referred to the time to disease progression, recurrence, transformation or death. POD24 was defined as confirmed disease progression within 24 months after frontline treatment, assessed based on the 2014 Lugano response criteria. Unified follow-up was conducted regularly, and the final follow-up cutoff date was December 31, 2022.

### 2.7 Statistical analysis

Categorical variables were compared using χ² or Fisher’s exact test. Continuous variables were analyzed using the Mann-Whitney U test. Survival curves were plotted by the Kaplan-Meier method and compared using log-rank and Wilcoxon tests. Univariate and multivariate Cox proportional hazards regression models were used to screen independent prognostic factors. Variables with missing values were excluded on an analysis-by-analysis basis in univariate Cox analyses. Only variables significant (p<0.05) in univariate analyses were entered into multivariate models. Time-dependent ROC curves and Harrell’s C-index were used to quantify the predictive performance of different prognostic models. Interaction analyses between SII and FLIPI components were performed using Cox proportional hazards regression models with interaction terms, implemented with the survival package in R. All statistical tests were two-sided, and P< 0.05 was considered statistically significant. All analyses were performed using SPSS 26.0 and R 4.4.1.

## 3 Results

### 3.1 Baseline characteristics

A total of 628 patients with grade 1–3a FL were included, including 431 in the training cohort and 197 in the validation cohort. Baseline clinicopathological characteristics were well balanced between the two cohorts (all P > 0.05; **Table 1**). The median age of the overall population was 53 years, and 297 (47.3%) patients were male. A total of 76.8% of patients presented with advanced Ann Arbor stage, and 75.3% received rituximab-based frontline immunochemotherapy. Notably, among the 473 patients undergoing frontline immuno-chemotherapy, the treatment backbones exhibited a near-universal distribution, with 465 patients (98.3%) receiving the R-CHOP regimen and only 8 patients (1.7%) receiving the R-CVP regimen. The median follow-up time was 38 months. The baseline differences between low-SII and high-SII groups are summarized in **Table 2**.

**Table 1:** Baseline characteristics of patients in the training, internal validation, and external validation cohorts.

Characteristics	Training/internal cohort total (N = 628)	Training cohort (N = 431)	Internal validation cohort (N = 197)	External validation cohort (N = 187)	P value*	P value†
Age (years)					0.546	0.178
< 60	450 (72%)	312 (72%)	138 (70%)	125 (67%)		
≥ 60	178 (28%)	119 (28%)	59 (30%)	62 (33%)		
Gender					0.752	>0.9999
Male	297 (47%)	202 (47%)	95 (48%)	88 (47%)		
Female	331 (53%)	229 (53%)	102 (52%)	99 (53%)		
Ann Arbor					0.614	0.358
I-II	107 (17%)	72 (17%)	35 (18%)	37 (20%)		
III-IV	483 (77%)	337 (78%)	146 (74%)	141 (75%)		
NA	38 (6%)	22 (5%)	16 (8%)	9 (5%)		
ECOG					0.707	0.095
< 2	590 (94%)	409 (95%)	181 (92%)	171 (91%)		
≥ 2	8 (1%)	5 (1%)	3 (2%)	6 (3%)		
NA	30 (5%)	17 (4%)	13 (7%)	10 (5%)		
SII group					0.175	0.145
Low SII	426 (68%)	285 (66%)	141 (72%)	112 (60%)		
High SII	202 (32%)	146 (34%)	56 (28%)	75 (40%)		
B-symptoms					0.899	0.519
Absent	510 (81%)	353 (82%)	157 (80%)	155 (83%)		
Present	89 (14%)	61 (14%)	28 (14%)	22 (12%)		
Unknown	29 (5%)	17 (4%)	12 (6%)	10 (5%)		
Marrow Inv.					0.912	0.389
No	538 (86%)	370 (86%)	168 (85%)	155 (83%)		
Yes	88 (14%)	60 (14%)	28 (14%)	31 (17%)		
Unknown	2 (0%)	1 (0%)	1 (1%)	1 (0%)		
Extranodal Inv.					0.686	0.155
Yes	388 (62%)	264 (61%)	124 (63%)	103 (55%)		
No	240 (38%)	167 (39%)	73 (37%)	84 (45%)		
FLIPI score					0.480	0.386
0-1 (Low)	149 (24%)	104 (24%)	45 (23%)	37 (20%)		
2 (Int)	226 (36%)	150 (35%)	76 (39%)	64 (34%)		
3-5 (High)	215 (34%)	154 (36%)	61 (31%)	76 (41%)		
NA	38 (6%)	23 (5%)	15 (8%)	10 (5%)		
FLIPI2 score					0.669	0.325
0-1 (Low)	218 (35%)	151 (35%)	67 (34%)	60 (32%)		
2 (Int)	310 (49%)	208 (48%)	102 (52%)	94 (50%)		
3-5 (High)	72 (11%)	52 (12%)	20 (10%)	31 (17%)		
NA	28 (4%)	20 (5%)	8 (4%)	2 (1%)		
LDH elevated					0.365	0.084
Elevated	115 (18%)	83 (19%)	32 (16%)	25 (13%)		
Normal	513 (82%)	348 (81%)	165 (84%)	162 (87%)		
B2M elevated					0.355	0.440
Elevated	197 (31%)	130 (30%)	67 (34%)	50 (27%)		
Normal	430 (68%)	300 (70%)	130 (66%)	137 (73%)		
Unknown	1 (0%)	1 (0%)	0 (0%)	0 (0%)		
CRP elevated					0.705	0.476
Elevated	8 (1%)	5 (1%)	3 (2%)	4 (2%)		
Normal	495 (79%)	344 (80%)	151 (77%)	160 (86%)		
Unknown	125 (20%)	82 (19%)	43 (22%)	23 (12%)		
Bulky mass					0.260	0.818
Yes	109 (17%)	80 (19%)	29 (15%)	32 (17%)		
No	476 (76%)	323 (75%)	153 (78%)	139 (74%)		
NA	43 (7%)	28 (6%)	15 (8%)	16 (9%)		
First-line treatment					0.167	<0.001
R-based	473 (75%)	333 (77%)	140 (71%)	187 (100%)		
Chemo	151 (24%)	96 (22%)	55 (28%)	0 (0%)		
Others	4 (1%)	2 (0%)	2 (1%)	0 (0%)		

Data are presented as n (%). The Total column represents the primary cohort, including the training and internal validation cohorts, and does not include the external validation cohort. Percentages were calculated using the corresponding cohort size and may not sum to 100% because of rounding. NA or Unknown categories indicate missing or unavailable data and were excluded from statistical comparisons. P Value* compares the training cohort with the internal validation cohort, whereas P Value† compares the training cohort with the external validation cohort. Two-sided Pearson’s chi-square tests or Fisher’s exact tests were used as appropriate when expected cell counts were small. Abbreviations: LDH, lactate dehydrogenase; CRP, C-reactive protein; β2M, β2-microglobulin; FLIPI, Follicular Lymphoma International Prognostic Index; R-based, rituximab-based therapy; SII, systemic immune-inflammation index.

**Table 2:** Correlations between baseline SII and clinicopathological characteristics in training and validation cohorts.

Characteristic	Training cohort (n=431)			Validation cohort (n=197)		
	Low SII (≤ 580)	High SII (>580)	*P* value	Low SII (≤ 580)	High SII (>580)	*P* value
No. of patients	285	146		141	56	
Age≥60	75	44	*0.401*	40	19	*0.442*
Male sex	139	63	*0.268*	69	26	*0.751*
Ann Arbors stage III-IV	219	118	*0.344*	105	41	*0.856*
B symptoms	42	19	*0.627*	22	6	*0.375*
ECOG≥2	2	3	*0.342*	1	2	*0.195*
Elevated LDH	44	39	*0.005*	18	14	*0.036*
Elevated B2M	90	40	*0.371*	43	16	*0.790*
Elevated CRP	3	2	*>0.999*	2	1	*0.522*
Marrow Inv.	48	12	*0.014*	25	3	*0.025*
Extranodal Inv.	113	54	*0.591*	52	21	*0.935*
Bulky mass	42	38	*0.004*	17	12	*0.094*
FLIPI risk category			*0.0095*			*0.327*
Low	80	24		35	10	
Intermediate	99	51		50	26	
High	94	60		45	16	
FLIPI2 risk category			*0.418*			*0.653*
Low	104	47		50	17	
Intermediate	132	76		70	32	
High	37	15		15	5	

Patients were stratified into low SII (≤ 580) and high SII (> 580) groups according to the optimal cutoff value. Correlations between SII status and baseline clinicopathological features were analyzed.

### 3.2 Optimal SII cutoff identification

**Figure 2:**
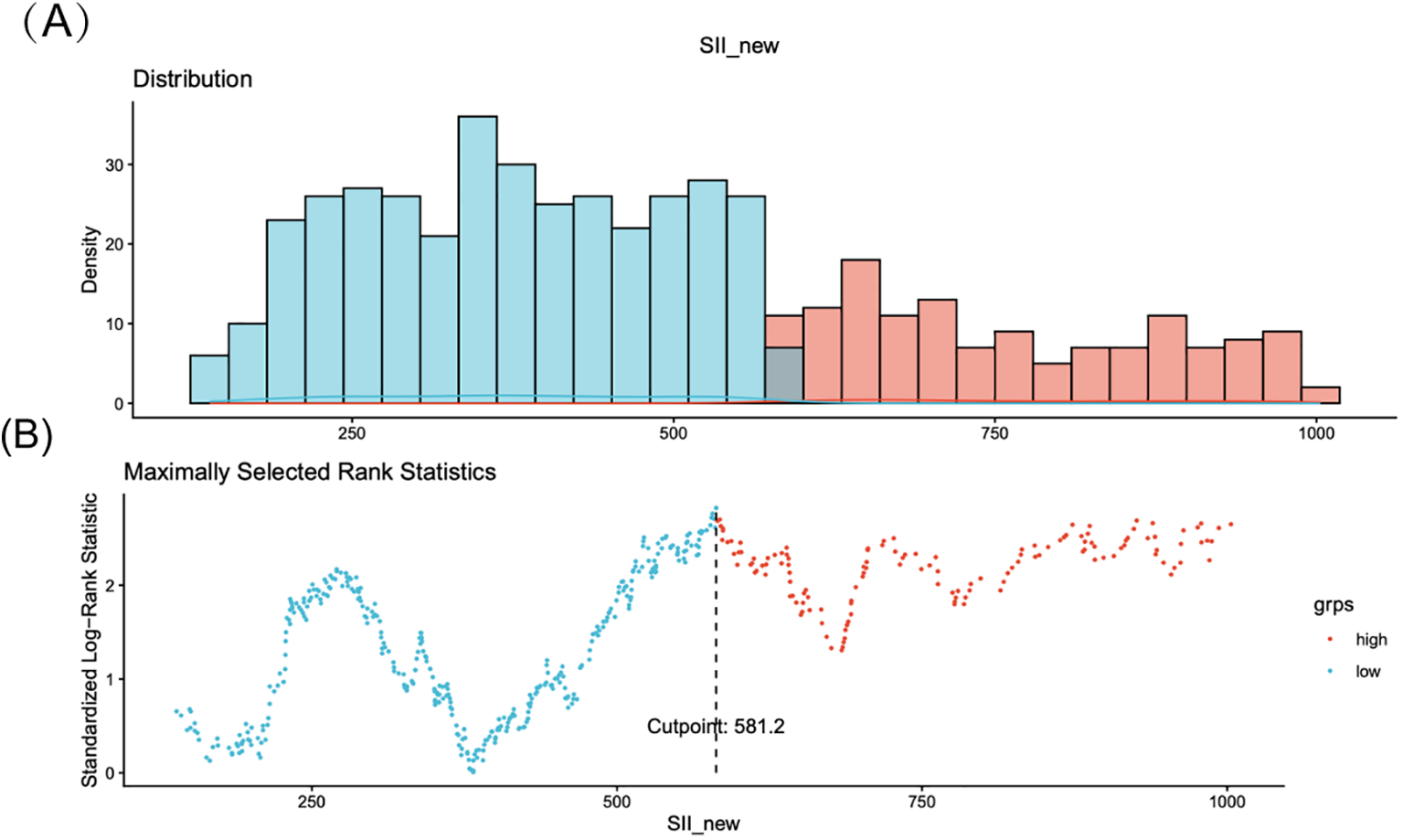
Determination of the optimal cutoff value of baseline systemic immune-inflammation index (SII). **(A)** Distribution histogram of baseline SII values. **(B)** Maximally selected rank statistics showing the optimal cutoff value of 581.2.

SII showed a right-skewed distribution. The optimal cutoff value was 581.2, which was rounded to 580 for ease of clinical application. Because the raw and rounded cutoff values were almost identical, 580 was used as the final cutoff in subsequent analyses (**Supplementary Figure 1**). We further assessed the association between SII and survival risk by plotting HR curves across different SII cut-off values. The HRs for both OS and PFS tended to increase when the SII cut-off exceeded 580, suggesting that higher SII levels were associated with poorer survival outcomes (**Supplementary Figure 2**). This threshold exhibited the best prognostic stratification for PFS and was significantly associated with OS (**Figure 2**). Based on this cutoff, 285 (66.1%) patients were classified into the low-SII group, and 146 (33.9%) into the high-SII group.

### 3.3 Prognostic significance of SII in training and validation cohorts

**Figure 3:**
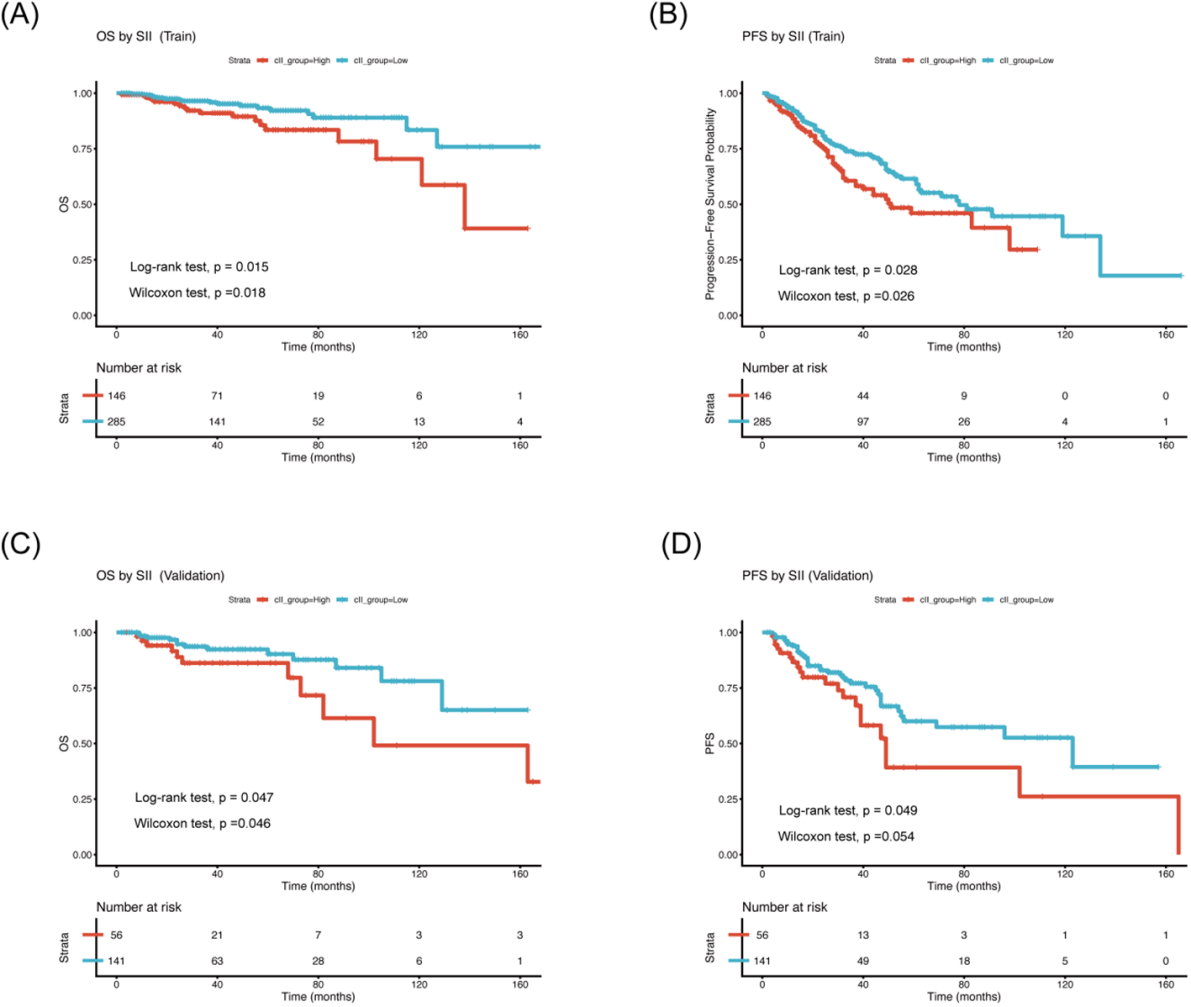
Kaplan–Meier survival curves of OS and PFS stratified by SII in the training and validation cohorts. In the training cohort, significant differences in OS (P = 0.015) and PFS (P = 0.028) were observed between the high-SII (> 580) and low-SII (≤ 580) groups **(A, B)**. Independent validation confirmed consistent prognostic differences for OS (P = 0.047) and PFS (P = 0.049) in the validation cohort **(C, D)**. The numbers at risk are listed below each panel.

In the training cohort, patients with high SII exhibited significantly inferior OS (log-rank P = 0.015; Wilcoxon P = 0.018) and PFS (log-rank P = 0.028; Wilcoxon P = 0.026) compared with those with low SII (**Figures 3A, B**).

These findings were validated in the independent validation cohort: high SII was associated with shorter OS (log-rank P = 0.047; Wilcoxon P = 0.046) and PFS (log-rank P = 0.049; Wilcoxon P = 0.054) (**Figures 3C, D**).

### 3.4 Independent prognostic value of SII

**Figure 4:**
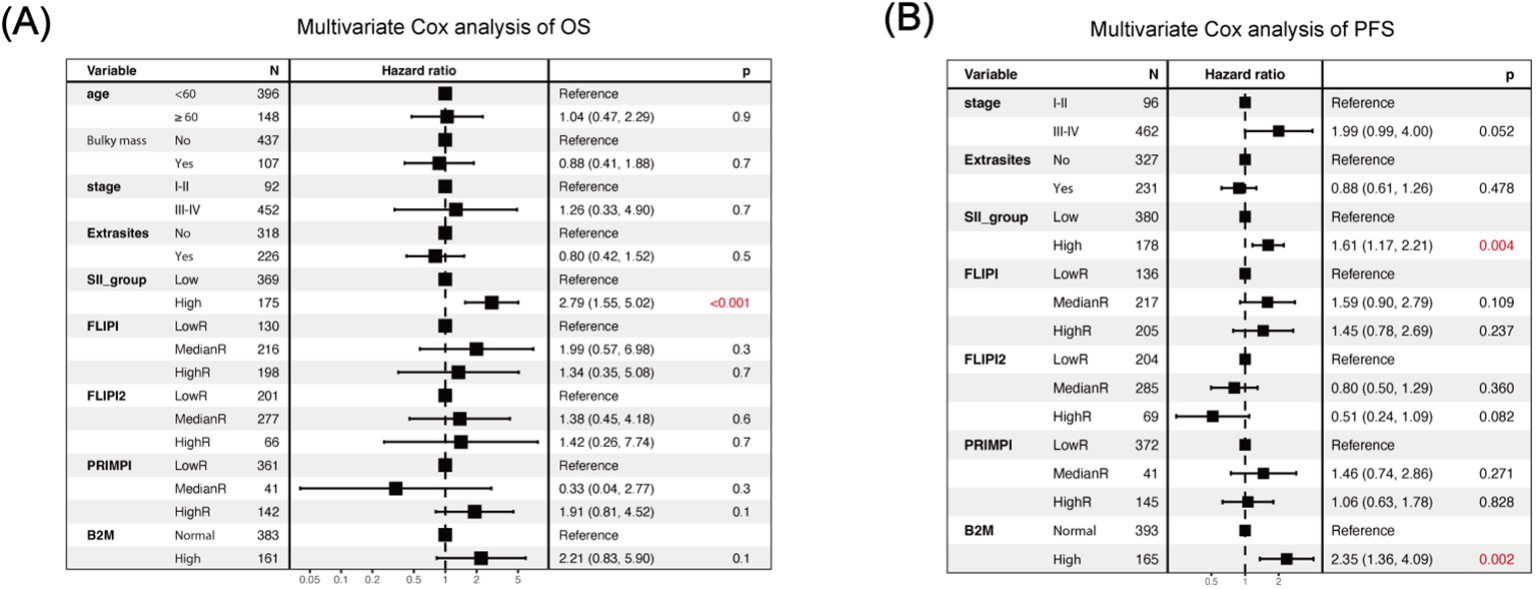
Forest plots of multivariate Cox regression analysis for independent prognostic factors of OS and PFS. **(A)** Multivariate Cox analysis of overall survival (OS). **(B)** Multivariate Cox analysis of progression-free survival (PFS).

Univariate Cox regression analysis identified high SII, age, stage, maximum tumor diameter, Extrasites, FLIPI, FLIPI2, PRIMPI and β2-microglobulin as significant predictors of poor OS. In the multivariate model, high SII remained an independent adverse prognostic factor for OS (HR = 2.79, 95%CI: 1.55–5.02, *P*<0.001) and PFS (HR = 1.61, 95%CI: 1.17–2.21, *P* = 0.004) (**Figures 4A, B**).

### 3.5 Subgroup analysis in rituximab-treated patients

**Figure 5:**
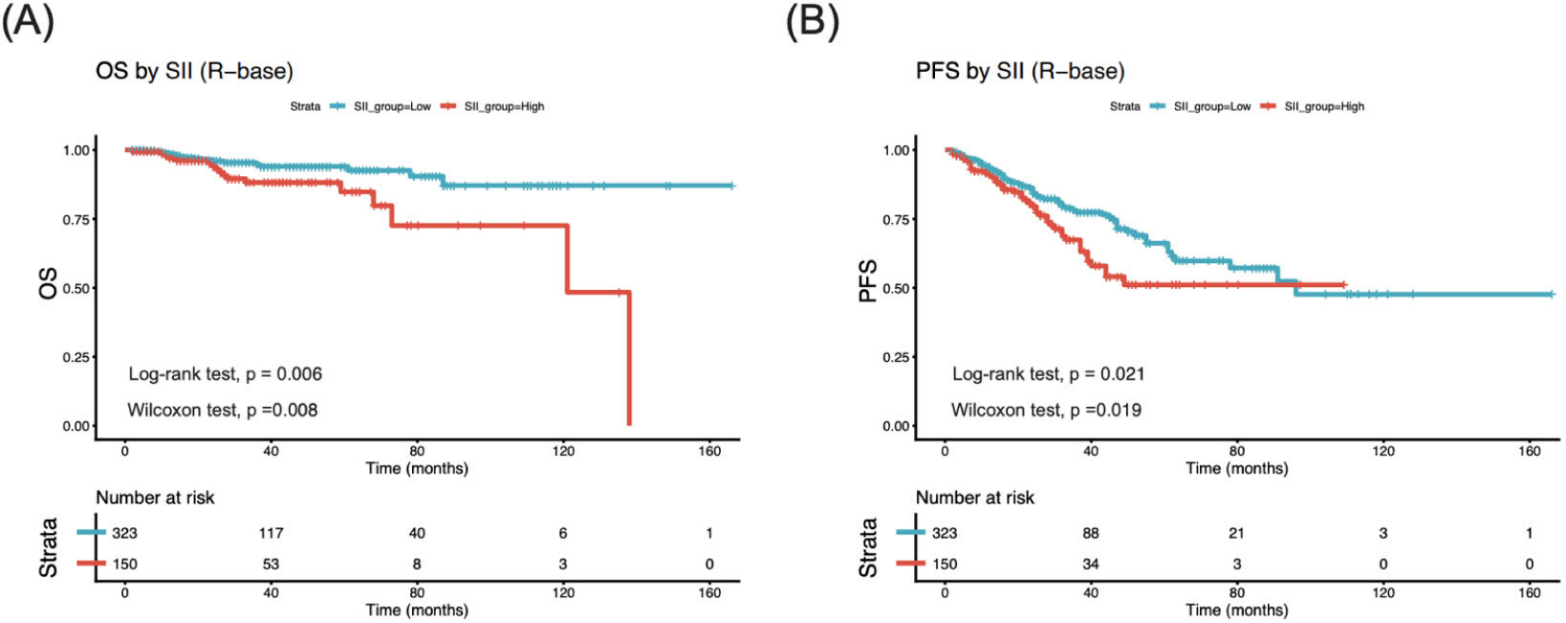
Prognostic value of SII in the rituximab-based therapy subgroup. **(A)** Kaplan-Meier curve of overall survival (OS) by SII group. **(B)** Kaplan-Meier curve of progression-free survival (PFS) by SII group.

Among 473 patients receiving rituximab-based frontline therapy, high SII remained significantly associated with inferior OS (log-rank *P* = 0.006; Wilcoxon *P* = 0.008) and PFS (log-rank *P* = 0.021; Wilcoxon *P* = 0.019) (**Figure 5**). This confirms the prognostic relevance of SII in the modern immunotherapy era.

### 3.6 Risk stratification ability of FLIPI-SII model

**Figure 6:**
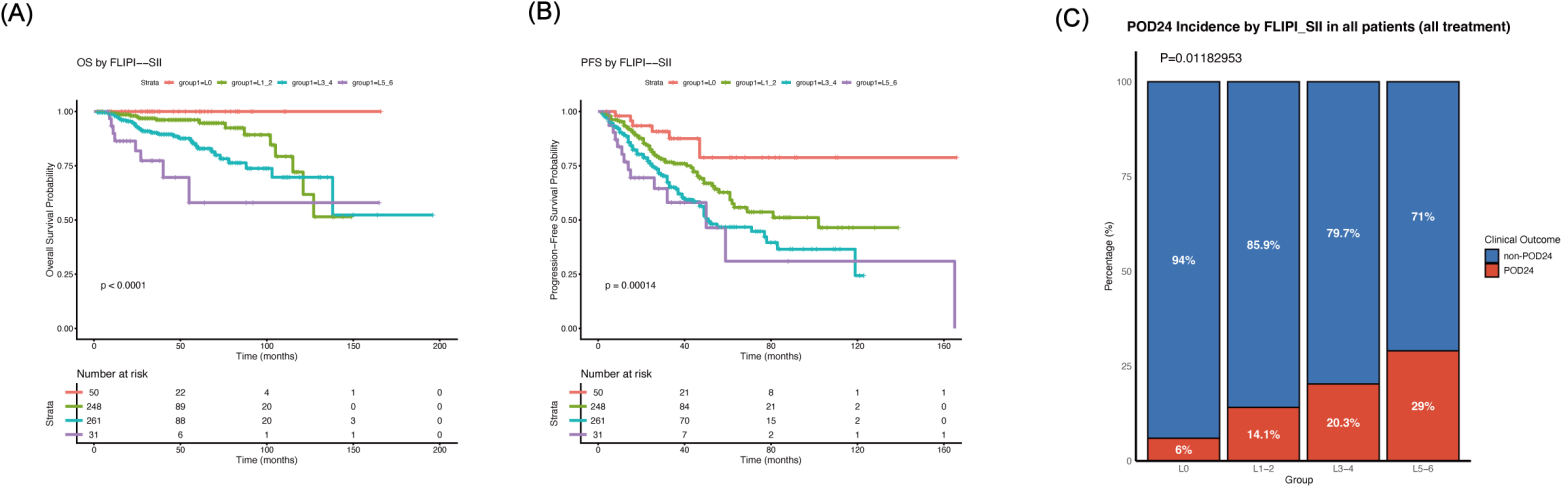
Risk stratification efficiency of the FLIPI-SII model for OS, PFS, and POD24. **(A)** Kaplan-Meier curve for overall survival (OS) probability by FLIPI-SII group. **(b)** Kaplan-Meier curve for progression-free survival (PFS) by FLIPI-SII group. **(c)** Bar chart comparing POD24 incidence percentages by FLIPI-SII group.

The FLIPI-SII model effectively divided patients into four risk subgroups with significantly different survival outcomes. Patients in the high-risk subgroups (L3–4, L5–6) presented remarkably shorter OS (P< 0.0001) and PFS (P = 0.00014) (**Figures 6A, B**).

To further justify the point-based FLIPI-SII scoring system, we constructed a Cox regression-based nomogram for OS prediction. High SII contributed substantially to the total points in the nomogram, and except for the number of involved nodal sites, the point contributions of all other variables were not lower than 50 points. These findings support the inclusion of SII as an additional 1-point adverse factor in the FLIPI-SII model, while maintaining the simplicity and clinical applicability of the original FLIPI framework (**Supplementary Figure 3**).

To evaluate the potential impact of treatment-era heterogeneity, we performed a sensitivity analysis stratified by enrollment period (2006–2015 vs. 2016–2020). FLIPI-SII retained prognostic discrimination for OS and PFS in both periods, supporting the robustness of the model across evolving treatment paradigms (**Supplementary Figure 4**).

### 3.7 Prognostic value of FLIPI-SII for POD24

Although single SII could not independently predict POD24 (P = 0.135), the combined FLIPI-SII model showed powerful predictive efficiency. The POD24 incidence increased gradually from 6.0% (L0 group) to 29.0% (L5–6 group) (P = 0.011; **Figure 6C**). Interaction analyses showed no significant interactions between high SII and individual FLIPI components for POD24 prediction, including age >60 years, Ann Arbor stage III/IV, hemoglobin<120 g/L, >4 nodal areas, and elevated LDH, with all P values for interaction >0.05(**Supplementary Table 1**).

### 3.8 Comparison of prognostic model performance

**Figure 7:**
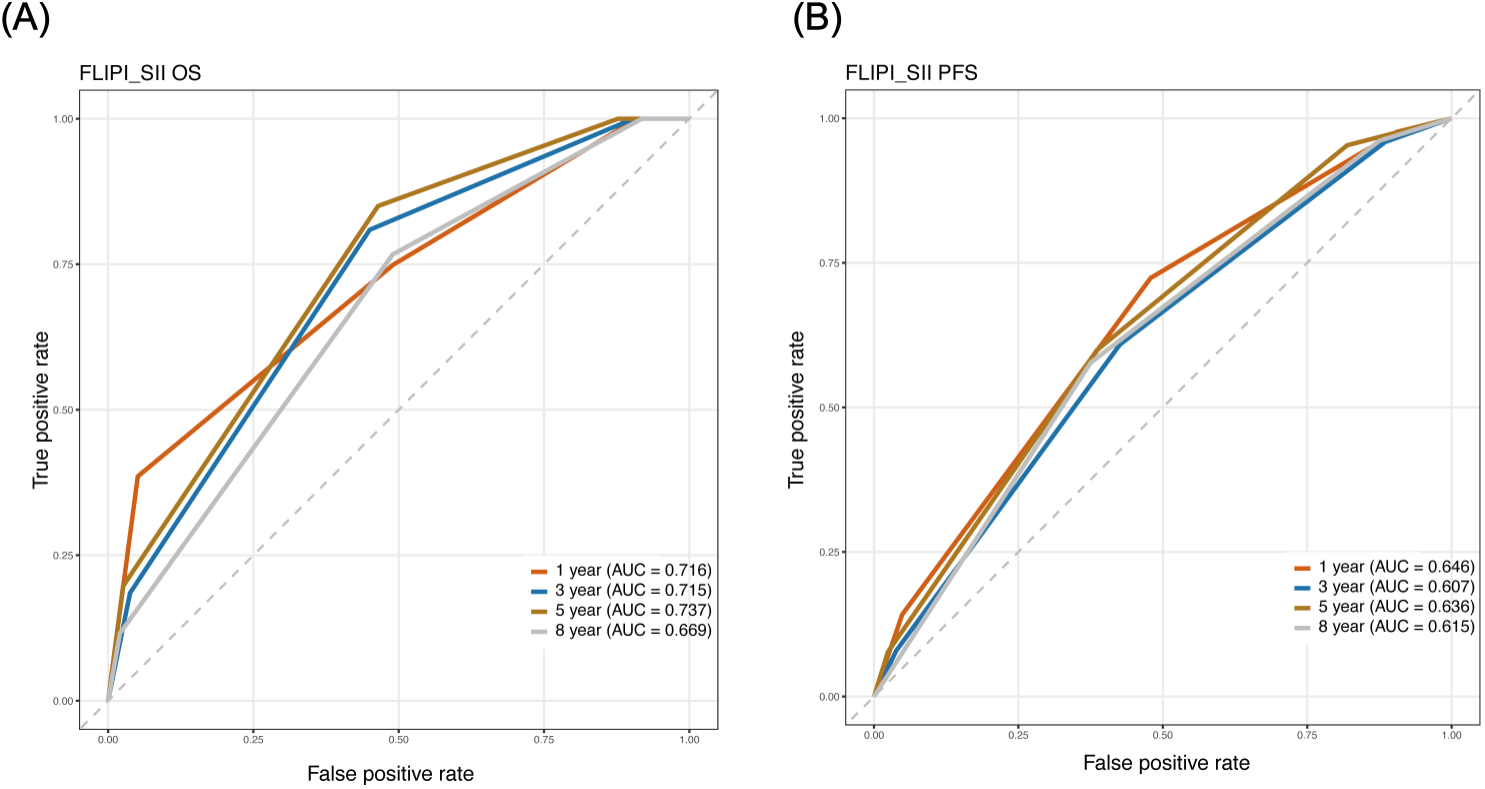
Time-dependent ROC curves of the FLIPI-SII model for 1-8-year survival prediction. **(A)** ROC curves comparing prediction performance for OS. **(B)** ROC curves comparing prediction performance for PFS.

Time-dependent ROC analysis demonstrated that the FLIPI-SII model achieved favorable predictive accuracy for PFS and OS (**Figure 7**). The AUCs for PFS ranged from 0.607 to 0.646 across 1–8 years, while the AUCs for OS ranged from 0.669 to 0.737, peaking at 5 years (AUC = 0.737). Calibration plots were further generated to evaluate the agreement between predicted and observed outcomes (**Supplementary Figure 5**).

The discriminative ability of the FLIPI-SII model was compared with conventional prognostic indices using Harrell’s C-index (**Table 3**). The FLIPI-SII model achieved superior predictive accuracy for both OS and PFS: OS C-index: 0.696 (FLIPI-SII) vs 0.640 (FLIPI), 0.642 (FLIPI2), 0.683 (PRIMPI); PFS C-index: 0.594 (FLIPI-SII) vs 0.589 (FLIPI), 0.546 (FLIPI2), 0.588 (PRIMPI) (**Table 3**). Previous studies have suggested that FLIPI performs better in predicting OS, whereas PRIMPI shows superior performance in predicting PFS. Accordingly, we compared FLIPI-SII with FLIPI for OS to assess the additional prognostic value of FLIPI-SII. Similarly, we compared FLIPI-SII with PRIMPI for PFS to evaluate whether FLIPI-SII offered improved predictive performance for PFS. NRI and IDI analyses were performed. Compared with the FLIPI model for OS prediction, FLIPI-SII showed improved reclassification and discrimination at 3 years (NRI = 0.245, p = 0.034; IDI = 0.013, p = 0.028), and maintained a favorable trend at 5 years (NRI = 0.247, p = 0.050; IDI = 0.018, p = 0.096). For PFS prediction, FLIPI-SII also outperformed PRIMPI at both 3 years (NRI = 0.183, p< 0.001; IDI = 0.017, p = 0.016) and 5 years (NRI = 0.202, p = 0.002; IDI = 0.038, p = 0.002) (**Supplementary Table 2**). These findings indicate that incorporating SII into the prognostic model provides additional predictive value beyond established clinical indices.

**Table 3:** Comparison of Harrell’s C-index among different prognostic models for follicular lymphoma.

Model	C-index	95%CI	C-index	95%CI
	OS		PFS	
FLIPI-SII	0.696	0.628-0.763	0.594	0.552-0.635
FLIPI	0.640	0.575-0.705	0.589	0.549-0.629
FLIPI-2	0.642	0.563-0.721	0.546	0.504-0.588
PRIMPI	0.683	0.614-0.751	0.588	0.548-0.628

Harrell’s C-index was used to compare the discriminatory performance of the FLIPI-SII model with conventional FLIPI, FLIPI2, and PRIMPI models for overall survival (OS) and progression-free survival (PFS).

These results demonstrate that integrating SII with the FLIPI index improves prognostic stratification beyond that achieved by conventional models alone.

### 3.9 External validation of the FLIPI-SII model

We further validated the FLIPI-SII model in an independent external multicenter cohort of 187 R-CHOP-treated FL patients from two additional Chinese hospitals. As summarized in **Table 1**, all essential baseline clinicopathological characteristics—including age, sex, tumor stage, and FLIPI/FLIPI2 risk groups—were well-balanced and highly comparable between the training and external validation cohorts (all p > 0.05). The only expected divergence was frontline treatment (p< 0.001), owing to the inclusion of a homogenous cohort receiving 100% R-CHOP in the external centers.

**Figure 8:**
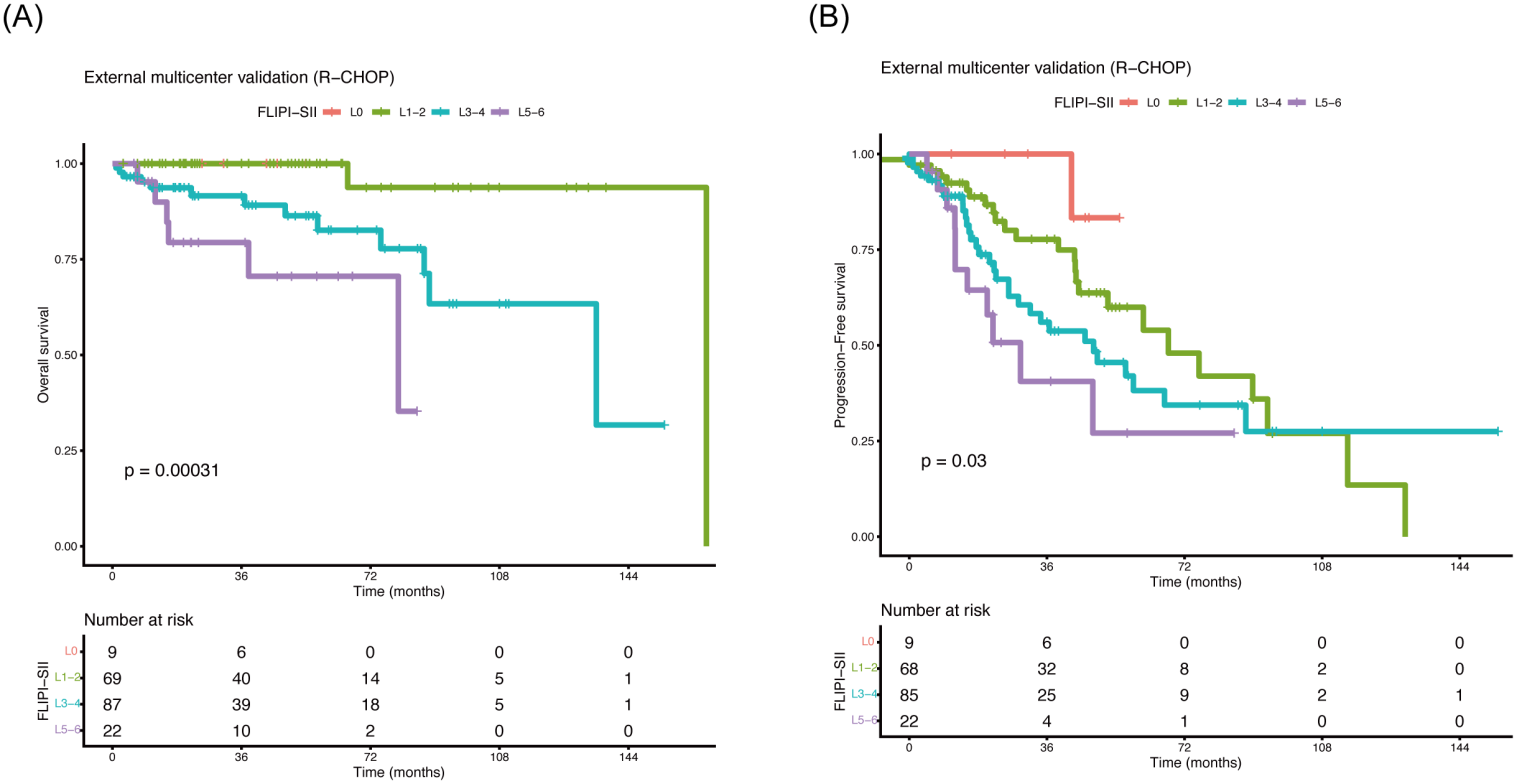
External multicenter validation of the FLIPI-SII model in 187 R-CHOP-treated patients with follicular lymphoma. **(A)** Overall survival. **(B)** Progression-free survival.

In this external cohort, the FLIPI-SII model retained significant prognostic stratification ability. Patients with higher FLIPI-SII scores had markedly inferior OS and PFS compared with those with lower scores. The log-rank test showed significant differences across FLIPI-SII risk groups for both OS and PFS (p=0.00031 for OS, and p=0.03 for PFS) (**Figure 8**). These findings indicate that the FLIPI-SII model remained robust in an independent multicenter validation cohort.

### 3.10 Head-to-head comparison with the FLIPI24 index

To evaluate the comparative prognostic efficiency of the FLIPI-SII model against state-of-the-art stratification tools, a direct head-to-head analysis with the recently proposed FLIPI24 index was performed in a complete-case subset (*N* = 468, **Supplementary Figure 6**). As detailed in **Supplementary Table 3**, the continuous FLIPI24 risk model achieved superior accuracy for predicting long-term OS (Overall Survival), presenting a higher C-index than the integer-based FLIPI-SII framework. Crucially, however, for PFS—the essential proximal endpoint for disease transformation and management—the FLIPI-SII model demonstrated statistically equivalent predictive performance compared with FLIPI24. Time-dependent ROC analysis revealed no significant differences in PFS prediction between the two models at both 3 years (AUC: 0.650 vs. 0.620, *p* = 0.397) and 5 years (AUC: 0.608 vs. 0.632, *p* = 0.712). These data indicate that while FLIPI24 excels in long-term mortality tracking, FLIPI-SII provides highly comparable, non-inferior disease progression stratification.

## 4 Discussion

Based on a large-sample multicenter retrospective cohort with both internal and external validation, this study systematically clarified that baseline peripheral blood SII is a stable, non-invasive and independent prognostic biomarker for patients with grade 1–3a FL. Elevated SII indicates poor long-term OS and PFS, and its predictive value is still reliable in patients receiving rituximab-based immunochemotherapy. More importantly, we innovatively constructed a FLIPI-SII combined prognostic model integrating immune-inflammatory indicators with traditional clinical parameters. Compared with classic FLIPI, FLIPI2 and PRIMPI, this novel system significantly improves the predictive accuracy of POD24 and long-term survival, and complements the latest FLIPI24 blood biomarker-oriented prognostic system, providing a new optimization direction for refined risk stratification of FL in the current immunotherapy era.

The prognostic value of SII in FL can be attributed to its reflection of the systemic immune-inflammation balance, a key determinant of tumor progression and immune therapy response (21). High SII is characterized by elevated neutrophils and platelets, which promote tumor growth through multiple mechanisms: neutrophils secrete pro-inflammatory cytokines (e.g., IL-6, TNF-α) and matrix metalloproteinases (MMPs) that enhance angiogenesis and tissue invasion, while platelets release growth factors (e.g., PDGF, VEGF) and form a physical barrier around tumor cells to evade immune recognition (22, 23). Conversely, reduced lymphocyte counts impair antitumor immune surveillance, including the activity of cytotoxic T cells and natural killer (NK) cells that are critical for eliminating malignant B cells (13, 24, 25). In FL, a TME-dependent lymphoma, this pro-tumor inflammatory state likely exacerbates immune escape and therapy resistance, leading to inferior outcomes.

A clinically meaningful finding is that SII retains prognostic significance in patients treated with rituximab-based therapy. Rituximab exerts its efficacy primarily through ADCC, complement-dependent cytotoxicity (CDC), and direct induction of apoptosis (26, 27). Plausibly, a high-SII environment may mirror a state of compromised immune effector function. It is reasonable to hypothesize that chronic systemic inflammation and platelet-derived anti-inflammatory mediators could collectively attenuate host innate immunity, thereby undermining the systemic immune fitness required for optimal rituximab-mediated clearance. While direct translational data regarding specific molecular cascades—such as NK-cell exhaustion phenotypes (28, 29), Fcγ receptor alterations, or platelet-derived TGF-β-mediated signaling pathways (30, 31)—were not explicitly evaluated in our retrospective cohort, these established immunological mechanisms provide a possible theoretical framework for our findings. The observed clinical correlation implies that a systemic pro-inflammatory state may serve as an effective peripheral surrogate for microenvironmental resistance to monoclonal antibody therapies. This clinical link highlights the potential of targeting systemic inflammation as a synergistic strategy to enhance the efficacy of immunotherapy in FL.

POD24 is a critical endpoint in FL, as it identifies patients at high risk of early mortality and transformation (32). Conventional prognostic indices, such as FLIPI, exhibit suboptimal accuracy in predicting POD24, typically yielding low C-index values (4). To address this prognostic gap, the recently proposed FLIPI24 model incorporated peripheral blood routine parameters, highlighting the clinical utility of minimally invasive blood biomarkers in modern risk stratification (20). When positioned directly against this newly introduced FLIPI24 index, our combined FLIPI-SII model exhibits distinct pragmatic and conceptual advantages. While the complex mathematical formulas of FLIPI24 deliver exquisite statistical performance for OS prediction, its baseline reliance on continuous multi-variable probabilities may restrict immediate, calculator-free bedside application. In contrast, FLIPI-SII operates as a straightforward 6-point risk score using zero-cost CBC-derived markers, making it seamlessly reproducible in global, resource-constrained clinical settings. Crucially, for progression-free survival (PFS)—the primary marker of clinical relapse in FL—the FLIPI-SII model yielded a statistically identical predictive performance to FLIPI24 in our cohort (*p* > 0.05), demonstrating its high statistical non-inferiority and pragmatic efficiency. Building upon these advancements, the FLIPI-SII model offers a complementary conceptual and biological framework. While models like FLIPI24 utilize individual hematological parameters, the SII mathematically integrates three distinct leukocyte lineages into a synchronized ratio. Rather than merely reflecting static raw cell counts, this composite index may capture the dynamic, homeostatic balance between pro-tumor chronic inflammation and host immunosurveillance within the follicular lymphoma macroenvironment. By coupling the tumor-centric burden metrics of the traditional FLIPI with the host-centric immune fitness captured by the SII, our combined model achieved a robust risk stratification for POD24, demonstrating a stepwise increase in POD24 incidence across distinct risk categories (from 6.0% in the lowest risk group to 29.0% in the highest risk group). This multi-dimensional integration potentially provides a more granular reflection of the host-tumor equilibrium, making it highly applicable for clinical risk assessment. Identifying these high-risk patients via the FLIPI-SII model might theoretically help guide early clinical counseling or strategic trial enrichment for novel microenvironment-targeted immunotherapies, such as bispecific antibodies, CAR-T cell therapies, or EZH2 inhibitors (33, 34).

The strengths of this study include its large sample size, training/validation cohort design, rigorous statistical methodology, and focus on clinically relevant endpoints. SII is derived from routine CBC tests, making it a low-cost, widely accessible biomarker that can be easily integrated into clinical practice—even in resource-limited settings.

**Limitations of this study** should be acknowledged: first, although our prognostic model was constructed and successfully verified utilizing an independent, multicenter external validation cohort to minimize institutional bias, the inherently retrospective design of the study may still introduce potential selection bias. Second, while the frontline treatment regimens remained relatively consistent (predominantly R-CHOP, 98.3%), the extreme imbalance in chemotherapy backbones (R-CHOP vs. R-CVP) precluded further multivariate stratification or adjustment from a statistical standpoint. Third, the median follow-up is relatively modest for an indolent disease like FL; while completely sufficient for a mature and robust POD24 assessment, the heavy censoring within this time window inevitably limits the maturity of long-term overall survival (OS) data and may constrain the extended evaluation of late-stage time-dependent ROC curves. Therefore, future prospective studies with longer follow-up are highly warranted to further validate the long-term performance of the FLIPI-SII model in broader clinical settings, particularly in the expanding era of chemotherapy-free regimens (e.g., rituximab plus lenalidomide), where therapeutic efficacy relies more intrinsically on host immune fitness. Additionally, further translational and mechanistic investigations are required to thoroughly dissect the exact cellular pathways and interactive cross-talk linking the peripheral SII to immune effector impairment. Such biological insights could formally guide the future development of novel combination strategies simultaneously targeting systemic chronic inflammation and immune checkpoints in follicular lymphoma.

## 5 Conclusion

In conclusion, baseline SII is an independent prognostic biomarker for survival in FL patients, and the FLIPI-SII combined model improves POD24 prediction. Integration of this simple, non-invasive biomarker into routine risk stratification may facilitate personalized immunotherapy strategies and improve outcomes in the rituximab era.

## Data Availability

The raw data supporting the conclusions of this article will be made available by the authors, without undue reservation.
