# Supplementary material for: Baseline systemic immune-inflammation index and a novel FLIPI-SII model predict POD24 and long-term survival in grade 1–3a follicular lymphoma
Source: Front Immunol. 2026 Jul 23;17:1869187. doi: 10.3389/fimmu.2026.1869187 (PMC13442641; doi:10.3389/fimmu.2026.1869187)
Supplement: Supplementary file 1 [file DataSheet1.docx]

Supplementary Materials

Supplementary Tables

| Interaction term | N | POD24_events | LR_χ² | df | P for interaction |
| --- | --- | --- | --- | --- | --- |
| SII_high × age >60 years | 628 | 109 | 0.352 | 1 | 0.553 |
| SII_high × stage III/IV | 590 | 98 | 0.052 | 1 | 0.82 |
| SII_high × hemoglobin <120 g/L | 626 | 109 | 0.19 | 1 | 0.663 |
| SII_high × >4 nodal areas | 619 | 106 | 0.242 | 1 | 0.623 |
| SII_high × elevated LDH | 628 | 109 | 0.034 | 1 | 0.855 |

Supplementary Table 1. Interaction analyses between SII and individual FLIPI components for POD24 prediction. Interaction effects between high SII and each FLIPI component were assessed using Cox proportional hazards regression models with multiplicative interaction terms. The likelihood ratio test was used to compare models with and without the interaction term. N indicates the number of patients included in each analysis; POD24_events indicates the number of patients experiencing progression of disease within 24 months; LR χ² indicates the likelihood ratio chi-square statistic; df indicates degrees of freedom. SII, systemic immune-inflammation index; FLIPI, Follicular Lymphoma International Prognostic Index; LDH, lactate dehydrogenase; POD24, progression of disease within 24 months.

| FLIPI | Endpoint | Time_point | NRI | NRI_95CI | NRI_P_value | IDI | IDI_95CI | IDI_P_value |
| --- | --- | --- | --- | --- | --- | --- | --- | --- |
|  | OS | 3-year | 0.245 | 0.000 to 0.466 | 0.034 | 0.013 | 0.000 to 0.054 | 0.028 |
|  | OS | 5-year | 0.247 | 0.000 to 0.455 | 0.05 | 0.018 | -0.002 to 0.075 | 0.096 |
| PRIMPI | Endpoint | Time_point | NRI | NRI_95CI | NRI_P_value | IDI | IDI_95CI | IDI_P_value |
|  | PFS | 3-year | 0.183 | 0.070 to 0.288 | <0.001 | 0.017 | 0.002 to 0.042 | 0.016 |
|  | PFS | 5-year | 0.202 | 0.066 to 0.335 | 0.002 | 0.038 | 0.008 to 0.085 | 0.002 |

Supplementary Table 2. Incremental predictive value of the FLIPI-SII model for overall survival and progression-free survival.

| **outcome** | **model** | **C_index(95%CI)** | **AUC_36m** | **AUC_60m** |
| --- | --- | --- | --- | --- |
| OS | FLIPI24 | 0.825(0.761-0.888) | 0.829 | 0.836 |
| OS | FLIPI-SII | 0.696(0.628-0.763) | 0.689 | 0.704 |
| PFS | FLIPI24 | 0.658(0.598-0.719) | 0.65 | 0.608 |
| PFS | FLIPI-SII | 0.594(0.552-0.635) | 0.62 | 0.632 |

Supplementary table 3. Predictive performance of FLIPI24 and FLIPI-SII for OS and PFS. Model performance was assessed by C-index and time-dependent AUCs at 36 and 60 months.

Supplementary Figures

Supplementary Figure 1: Distribution of the systemic immune-inflammation index, with the raw optimal cutoff value of 581.2 and the rounded cutoff value of 580 indicated.

Supplementary Figure 2: Hazard ratio curve for OS and PFS across different SII cutoff values.

Supplementary Figure 3. Cox regression-based nomograms for predicting survival outcomes using FLIPI-SII components.

(A) Nomogram for predicting 3-year and 5-year overall survival (OS) probability based on the six FLIPI-SII components, including age >60 years, Ann Arbor stage III/IV, low hemoglobin, involvement of >4 nodal areas, elevated LDH, and high SII.

(B) Nomogram for predicting 3-year and 5-year progression-free survival (PFS) probability based on the same six FLIPI-SII components.

Supplementary Figure 4. Prognostic performance of the FLIPI-SII model across different treatment eras. Kaplan–Meier curves for overall survival (OS) and progression-free survival (PFS) according to FLIPI-SII risk groups. (A, B) OS and PFS in patients from TMUCIH diagnosed between 2006 and 2015. (C, D) OS and PFS in patients from TMUCIH diagnosed between 2016 and 2020.

Supplementary Figure 5. Calibration assessment of the FLIPI-SII model. Calibration curve for 3-year OS, 3-year PFS prediction and POD24.

Supplementary Figure 6. Survival outcomes stratified by FLIPI24 risk groups.
Kaplan–Meier curves of OS (A) and PFS (B) according to FLIPI24 low-, intermediate-, and high-risk groups. Both OS and PFS differed significantly among the three groups by log-rank test. Numbers at risk are shown below each plot.
